# Protective effect of absent Hyrtl anastomosis on fetal prognosis in monochorionic twins: Report of 2 cases and literature review

**DOI:** 10.1097/MD.0000000000049359

**Published:** 2026-06-19

**Authors:** Miao Hu, Dan Zhang, Zhitao Zhang

**Affiliations:** a Department of Obstetrics and Gynecology, Shenyang Women’s and Children’s Hospital, Shenyang, Liaoning, China; b Shenyang Clinical Medical Research Center for Obstetrics and Gynecology, Shenyang Women’s and Children’s Hospital, Shenyang, Liaoning, China.

**Keywords:** Hyrtl anastomosis, monochorionic pregnancy, placental perfusion, selective fetal growth restriction

## Abstract

**Rationale:**

Hyrtl anastomosis is an intra-arterial shunt between umbilical arteries, which exerts a protective effect by balancing arterial pressure in singleton pregnancies. Its absence may be associated with adverse outcomes such as fetal growth restriction. However, its role in monochorionic twin pregnancies remains unclear. This study aims to explore the clinical significance of the absence of Hyrtl anastomosis in monochorionic twin pregnancies through placental perfusion analysis.

**Patient concerns:**

Case 1: 32-year-old primigravida with natural conception, diagnosed with monochorionic diamniotic (MCDA) twin pregnancy at 12 weeks’ gestation. Fetal growth discrepancy was found at 24 weeks; case 2: 30-year-old G2P0 woman with an in vitro fertilization-conceived MCDA twin pregnancy.

**Interventions:**

Biweekly regular prenatal examinations throughout pregnancy, serial ultrasound monitoring of fetal growth and blood flow (umbilical artery, middle cerebral artery, ductus venosus). The patient in case 1 received an elective cesarean section at 36 + 5 weeks’ gestation, while the patient in case 2 underwent the surgery at 37 weeks’ gestation.

**Diagnoses:**

Case 1:type I selective intrauterine growth restriction; case 2: uncomplicated MCDA twin pregnancy.

**Outcomes:**

Placental perfusion confirmed the absence of Hyrtl anastomosis in both cases (the larger fetus in case 1, and the smaller fetus in case 2). The newborns had good Apgar scores, and the short-term and medium-term prognoses were excellent.

**Lessons:**

The absence of Hyrtl anastomosis may be a protective factor in monochorionic twin pregnancies, which can offset the adverse effects caused by uneven placental share and vascular communicating anastomoses, thereby improving fetal prognosis.

## 1. Introduction

The incidence of complications in monochorionic twin pregnancies is significantly higher than that in singleton pregnancies and dichorionic twin pregnancies. Diseases such as selective intrauterine growth restriction (SIUGR), twin-twin transfusion syndrome (TTTS), and twin anemia-polycythemia sequence pose a serious threat to fetal health. Placental factors are the key causes of these complications, among which uneven placental share distribution and vascular anastomoses between fetuses (especially artery-to-artery [A-A] anastomoses and artery-to-vein [A-V] anastomoses) are the core inducing factors. Hyrtl anastomosis is a natural shunt structure of the umbilical arteries near the placental insertion site. In singleton pregnancies, it can balance the pressure of the 2 umbilical arteries and play a “safety valve” role, and its absence is associated with adverse fetal outcomes.^[1]^ However, studies on Hyrtl anastomosis in monochorionic twin pregnancies are extremely scarce, and only a few case reports suggest that its absence may have a protective effect. This study further verifies the protective effect of the absence of Hyrtl anastomosis on the prognosis of fetuses in monochorionic twin pregnancies through 2 cases confirmed by placental perfusion, providing a new reference for clinical prenatal management.

## 2. Case presentation

### Case 1: a case of type I SIUGR with absence of Hyrtl anastomosis in the larger fetus

**Figure 1.**
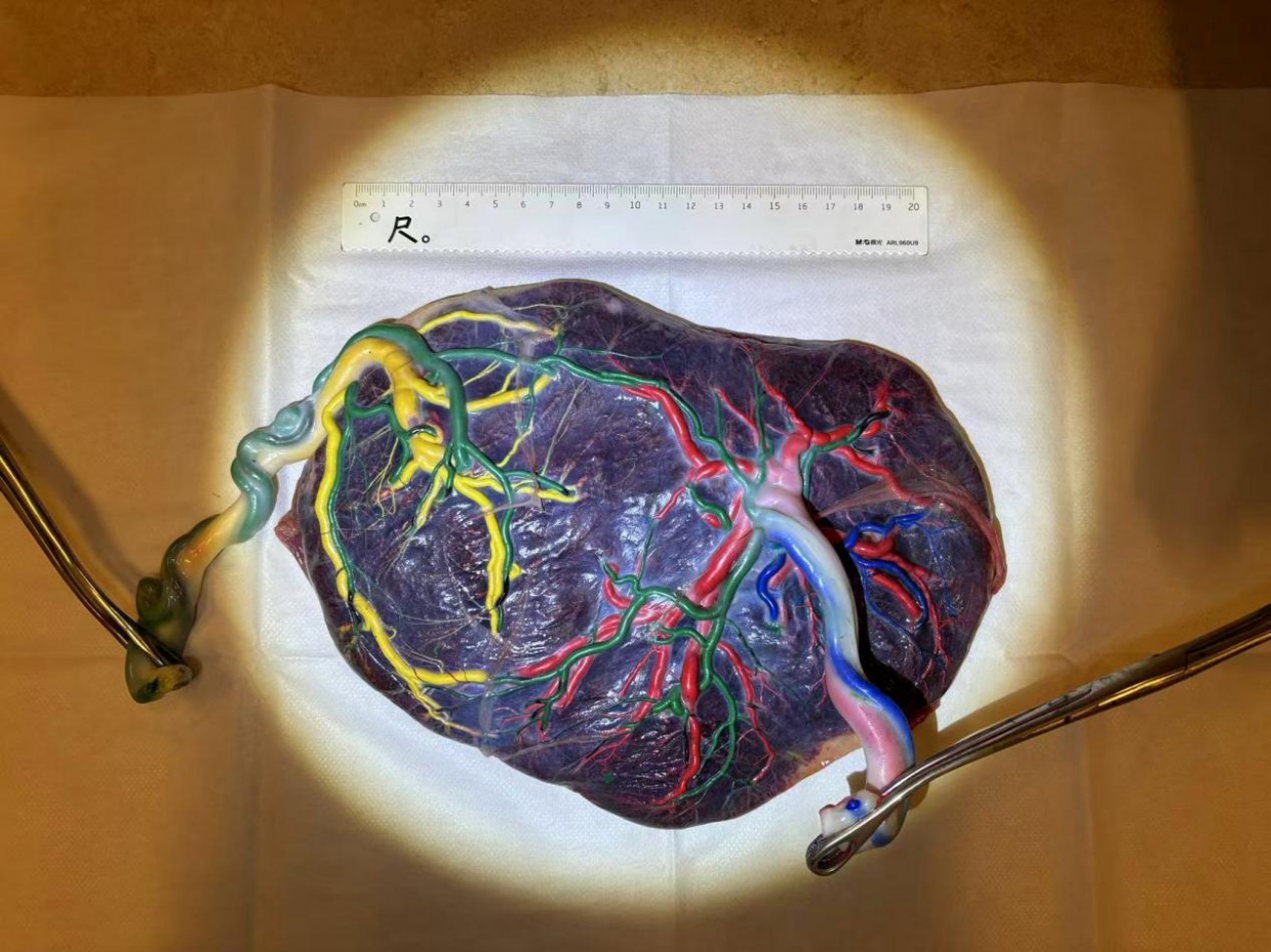
Placental perfusion staining results of case 1. Red dye was injected into the umbilical vein of the larger fetus, and yellow dye was injected into the umbilical vein of the smaller fetus; blue dye was injected into 1 umbilical artery of the larger fetus, with only 1 branch stained blue; green dye was injected into 1 umbilical artery of the smaller fetus, and green staining was observed in another umbilical artery branch of the larger fetus through the arterio-arterial (A-A) anastomosis; no dye mixing was found between the 2 umbilical arteries of the larger fetus, confirming the absence of Hyrtl anastomosis.

A 32-year-old primigravida conceived naturally. At 12 weeks of gestation, ultrasound confirmed a monochorionic diamniotic (MCDA) twin pregnancy, and regular prenatal examinations were performed every 2 weeks during pregnancy. At 24 weeks of gestation, ultrasound showed an estimated fetal weight difference of 20% between the twins. The weight of the smaller fetus was below the 3rd percentile for the same gestational age, the umbilical artery (UA) systolic/diastolic ratio was normal, the end-diastolic blood flow was positive, and the amniotic fluid volume was within the normal range, so type I SIUGR was diagnosed. Subsequent serial ultrasound monitoring showed that the growth rate of the twins was stable, and the blood flow indicators of UA, middle cerebral artery, and ductus venosus were normal throughout the pregnancy. At 36 + 5 weeks of gestation, an elective cesarean section was performed due to the persistent presence of SIUGR. Two male infants were delivered with birth weights of 2710 g and 1930 g, respectively. The birthweight discordance ratio was 29%. The 1-minute and 5-minute Apgar scores of the normal newborn were both 10, and the 1-minute and 5-minute Apgar scores of the smaller newborn were both 9 (skin color −1). The smaller newborn was admitted to the neonatal intensive care unit for observation for 5 days (without special treatment), and the growth indicators were normal at discharge. Follow-up at 12 months after birth showed no abnormalities in the physical and neurological development of the 2 newborns. Placental perfusion detection was performed using acrylic dye staining within 2 hours after delivery. The placenta was intact, and after flushing the blood vessels with normal saline, different colored dyes were injected into the umbilical veins and arteries of the twins, respectively. ImageJ software (National Institutes of Health, NIH) was used to measure the placental area: the total placental area was 345 cm^2^, the placental share of the larger fetus was 227 cm^2^ (65%), and that of the smaller fetus was 118 cm^2^ (35%), with a placental territory discordance ratio of 48%. Observation of dye distribution showed no dye mixing between the 2 umbilical arteries of the larger fetus, confirming the absence of Hyrtl anastomosis; there were thick A-A anastomoses, but no abnormal blood flow spectrum was observed by ultrasound during pregnancy (Fig. 1).

### Case 2: an uncomplicated MCDA case with absence of Hyrtl anastomosis in the smaller fetus

**Figure 2.**
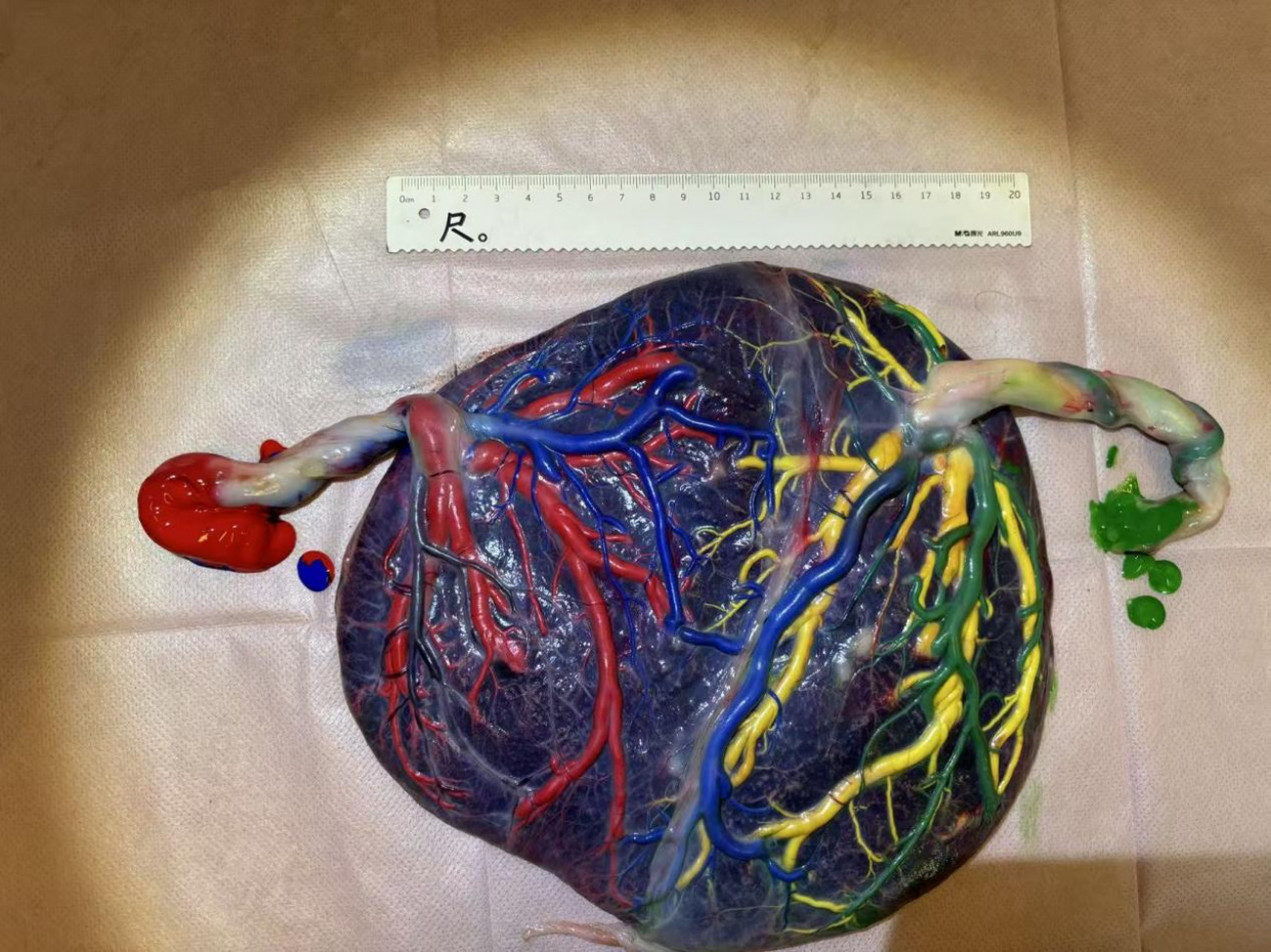
Placental perfusion staining results of case 2. Red and yellow dyes were injected into the umbilical veins of the 2 fetuses, respectively. Blue dye was injected into 1 umbilical artery of the smaller fetus, while the other umbilical artery showed no staining (absence of Hyrtl anastomosis). Then, blue staining was observed in the larger fetus through the arterio-arterial (A-A) anastomosis; green dye was injected into the umbilical artery of the larger fetus.

A 30-year-old pregnant woman, gravida 2, para 0, conceived by in vitro fertilization. Early pregnancy ultrasound confirmed an MCDA twin pregnancy, and noninvasive prenatal testing indicated a low risk. Regular prenatal examinations (once every 2 weeks) throughout the pregnancy showed that the amniotic fluid volume of the twins was normal, and the blood flow indicators of UA, middle cerebral artery, and ductus venosus were all normal. The estimated fetal weight of both fetuses remained at the 5th to 15th percentile for the same gestational age, with no significant growth difference. At 37 weeks of gestation, an elective cesarean section was performed at the request of the pregnant woman, and 2 female infants were delivered with birth weights of 2660 g and 2370 g, respectively, with a birthweight discordance ratio of 11%. The 1-minute and 5-minute Apgar scores of both newborns were 10, and there was no need for neonatal intensive care unit admission. Follow-up at 12 months after birth showed that the growth and development of the twins were normal. Postpartum placental perfusion staining showed that the total placental area was 429 cm^2^, the placental share of the larger fetus was 254 cm^2^ (59%), and that of the smaller fetus was 175 cm^2^ (41%). The placental territory discordance ratio was 31%. Dye injection results showed that there were thick A-A anastomoses between the fetuses; in addition, there was an absence of Hyrtl anastomosis in the smaller fetus (Fig. 2).

## 3. Conclusion

The common characteristics of the 2 cases are that both cases were MCDA twin pregnancies with an uneven distribution of placental territory (48%, 31%). Case 1 was complicated with type Ⅰ SIUGR, while case 2 had no complications. Placental perfusion confirmed the absence of Hyrtl anastomosis in both cases (the larger fetus in case 1, and the smaller fetus in case 2); no serious complications such as TTTS or severe SIUGR occurred during pregnancy, the newborns had good Apgar scores, and the short-term and medium-term prognoses were excellent.

## 4. Discussion

Hyrtl anastomosis is a small, transverse arterial channel that connects the 2 umbilical arteries before they ramify across the chorionic plate. It is present in approximately 90% to 95% of singleton placentas.^[1]^ First described by anatomist Joseph Hyrtl in 1870, this structure plays a crucial role in maintaining the balance and homeostasis of the fetal-placental circulation. As a hemodynamic balancer between the 2 umbilical arteries, it functions as a hemodynamic buffer and collateral circulation pathway when placental vascular resistance is asymmetric or lesions occur. In singleton pregnancies, its absence may be associated with adverse outcomes such as fetal growth restriction. However, the placental circulation mechanism of monochorionic twins differs from that of singletons. In previous studies, the analysis of the placenta regarding common special complications of monochorionic twins, such as SIUGR, was often attributed to higher placental territory discordance and the prevalence of thick A-A anastomoses.^[2]^ Few studies have analyzed the role of Hyrtl anastomosis in the placentas of monochorionic twins.

Literature review on Hyrtl anastomosis in monochorionic twin pregnancies, see Table 1: In 2 case reports by Liu et al,^[3,4]^ the placental characteristics of 4 cases of SIUGR were reported. Among them, the absence of the Hyrtl anastomosis was found in the fetus with a larger placental area in 3 cases. These 3 cases had a high placental territory discordance ratio (64.2%, 64.3%, 44.6%), but the Doppler blood flow of both fetuses was normal during pregnancy. Absent end-diastolic velocity was observed at 24 weeks in case 2, which returned to normal afterward, and the fetuses finally had a good prognosis. The other case was a monochorionic triamniotic pregnancy, confirmed at 12 weeks of gestation (fetus 1 was an acardiac fetus, which did not show significant volume increase during prenatal monitoring, and its UA blood flow gradually decreased to undetectable levels). At 16 weeks of gestation, severe growth restriction was revealed in fetus 3, associated with intermittent absent end-diastolic velocity. Cesarean section was performed at 34 weeks of gestation, and both fetuses had a good prognosis. Postpartum placental perfusion confirmed the absence of Hyrtl anastomosis in both fetus 2 and fetus 3. Walker^[5]^ reported 1 case of TTTS. This case represents an example of prolonged fetal survival associated with reversed end-diastolic flow (REDF). The time interval from diagnosis of REDF in the smaller twin to delivery was 15 days, and the persistence of REDF exceeded the time usually expected for such pathological findings. Anatomical examination of the placenta showed that, in addition to A-A anastomoses and A-V anastomoses (A to B), no Hyrtl anastomosis was found in twin A (donor). This vascular configuration may have led to different arterial pressures in the 2 umbilical arteries of twin A. The pressure difference between the 2 arteries may have allowed sufficient umbilical artery perfusion, thereby achieving adequate fetal oxygenation through blood flow in 1 umbilical artery (despite persistent REDF in the other umbilical artery). The reason for the prolonged disease course was the lack of a balancing anastomosis between the umbilical arteries, namely, Hyrtl anastomosis, which resulted in essentially 2 independent and separate placental circulations.

**Table 1:** Comparison of the overall condition and pregnancy outcomes

case	Author (Year)	complications of monochorionic twins	complication of pregnancy	umbilical artery Doppler flow	Gestational age at delivery, weeks	Delivery mode	Birthweight (large r/smaller)	Apgar (1minute 、5minute)	Placental territory discordance ratioa	absence of Hyrtl’s anastomosis
1	Liu Z. et al(2023)	SIUGR	Preterm labor	Normal	35 + 3	CS	2110/ 2000	10-/ 10-	64.2%	Larger
2	Liu Z. et al(2025)	SIUGR	Normal	AEDV at 23 weeks, then normal	35 + 5	CS	2410/ 1660	10-/ 10-	64.3%	Larger
3	Liu Z. et al(2025)	SIUGR	GDM	Normal	36 + 5	CS	2180/ 2300	10-/ 10-	44.6%	Larger
4	Liu Z. et al(2025)	F1acardiac F3SIUGR	chronic nephritis	16weeks intermittent AEDV	34	CS	1910/ 1370	9、10/ 9、10	12.7%	Both
5	Walker M.(1999)	TTTS、 SIUGR	Normal	26weeks REDV(donor)	28	CS	1230/ 515	6、9/ 8、9	Evaluate: 80%	Donor

AEDV = absent end-diastolic velocity, CS = cesarean section, F1 = fetus1, F3 = fetus3, GDM = gestational diabetes mellitus, REDV = reversed end-diastolic flow, SIUGR = selective intrauterine growth restriction, TTTS = twin-twin transfusion syndrome.

In the 2 cases of this study, in case 1, despite a 48% placental territory discordance ratio and the presence of thick A-A anastomoses, the absence of Hyrtl anastomosis in the larger fetus may have isolated the circulation of the 2 umbilical arteries. This not only prevented excessive blood loss from the larger fetus (as a potential “donor”) to the smaller fetus but also ensured adequate perfusion and oxygenation of the placental lobules of the larger fetus itself. Eventually, the birthweight discordance ratio was 29%, with no progression of SIUGR or other serious monochorionic twin complications. In case 2, to analyze the reasons why the smaller fetus did not develop complications, first, the placental territory discordance ratio (31%) may not have been significant; second, due to the absence of Hyrtl anastomosis, only 1 umbilical artery of the smaller fetus participated in the bidirectional A-A blood flow, resulting in relatively stable blood flow, thereby preventing the occurrence of complications such as SIUGR. Consistent with the speculation reported by Liu and Walker mentioned above, this further confirms that the absence of Hyrtl anastomosis may improve the prognosis of monochorionic twins with uneven placental share. A notable observation is that the absence of Hyrtl anastomosis is not confined to a specific fetal side relative to placental share – it can occur both in the fetus with a relatively larger placental territory and in the counterpart with a smaller placental allocation. Importantly, regardless of which fetal side this structural variation is located on, it exerts a consistent protective effect on the intrauterine development and hemodynamic stability of both fetuses.

These observations challenge the traditional focus on the pathological role of abnormal anastomoses in monochorionic twin complications and expand the understanding of Hyrtl anastomosis in fetal circulatory regulation.

The protective mechanism of the absence of Hyrtl anastomosis in monochorionic twins may be related to the unique vascular network of the monochorionic placenta. Inter-fetal vascular anastomoses (A-A anastomoses and A-V anastomoses) are relatively common in monochorionic twins, and the presence of Hyrtl anastomosis may increase the complexity of blood flow regulation, leading to shunt imbalance. Its absence can simplify the vascular regulation pathway and reduce the risk of excessive blood flow exchange between twins, thereby offsetting the adverse effects caused by an uneven placental share. This is essentially different from the pressure-balancing role of this structure in singleton pregnancies, reflecting the different adaptive mechanisms of placental vascular structures in different pregnancy types.

This study has limitations. First, as a case report, the sample size is small, and the conclusions need to be verified by large-sample prospective studies. Second, the precise hemodynamic and molecular mechanisms underlying this protective effect remain elusive. Further in vitro vascular modeling and in vivo hemodynamic monitoring studies are needed to elucidate the pathways through which Hyrtl anastomosis absence maintains circulatory stability. Third, prenatal evaluation of Hyrtl anastomosis was not performed. Future studies can explore the feasibility of imaging techniques such as ultrasound or magnetic resonance angiography for prenatal detection, focus on standardizing evaluations, incorporate them into risk prediction models, and investigate developmental control measures. Last, the long-term protective effect of the absence of Hyrtl anastomosis needs to be confirmed by longer-term follow-up.

In conclusion, the absence of Hyrtl anastomosis may be a key protective structural variation in monochorionic twin pregnancies, with its protective effect being consistent regardless of whether it is present on the larger or smaller placental share side. This finding provides new insights for prenatal risk stratification and prognostic evaluation of monochorionic twins and offers potential theoretical support for optimizing clinical management strategies and perinatal intervention protocols for such high-risk pregnancies.

## Acknowledgments

The authors would like to thank the patients for their cooperation with this study.

## Author contributions

**Data curation:** Miao Hu, Dan Zhang.

**Investigation:** Miao Hu.

**Methodology:** Miao Hu, Zhi-Tao Zhang.

**Supervision:** Dan Zhang.

**Writing – review & editing:** Zhi-Tao Zhang.

**Writing – original draft:** Miao Hu.
